# Supplementary material for: Associations of vaccine status with characteristics and outcomes of hospitalized severe COVID-19 patients in the booster era
Source: PLoS One. 2022 May 10;17(5):e0268050. doi: 10.1371/journal.pone.0268050 (PMC9089907; doi:10.1371/journal.pone.0268050)
Supplement: S1 Table — (DOCX) [file pone.0268050.s001.docx]

**Table S1, multivariate ordinal regression model for correlation of selected variables with vaccine status**

| Variable | Coefficient | | SE | 95% CI | Wald | p Value |
| --- | --- | --- | --- | --- | --- | --- |
| Age | | 0.033 | 0.008 | 0.017 to 0.049 | 16.89 | <0.001 |
| Male gender | | 0.787 | 0.242 | 0.313 to 1.262 | 10.59 | 0.001 |
| Number of comorbidities | | 0.168 | 0.061 | 0.048 to 0.288 | 7.57 | 0.006 |
| From symptoms to admission, days | | -0.076 | 0.027 | -0.13 to -0.023 | 7.77 | 0.005 |
| Hospital duration, days | | -0.01 | 0.018 | -0.046 to 0.026 | 0.316 | 0.574 |
| Critical disease | | 1.094 | 0.349 | 0.411 to 1.778 | 9.84 | 0.002 |
| Intubation | | -0.628 | 0.466 | -1.541 to 0.285 | 1.818 | 0.178 |
