# Supplementary material for: Associations of vaccine status with characteristics and outcomes of hospitalized severe COVID-19 patients in the booster era
Source: PLoS One. 2022 May 10;17(5):e0268050. doi: 10.1371/journal.pone.0268050 (PMC9089907; doi:10.1371/journal.pone.0268050)
Supplement: S2 Table — (DOCX) [file pone.0268050.s002.docx]

Table S2, Treatment during hospitalization and correlation to vaccine status.

|  | Total  n=349  No. (%) | No vaccine  n=202  No. (%) | Two Vaccines  n=122  No. (%) | Booster  n=25  No. (%) | P |
| --- | --- | --- | --- | --- | --- |
| Casirivimab plus imdevimabª | 52 (15) | 31 (15) | 17 (14) | 4 (16) | 0.930 |
| Remdesivir | 57 (16) | 30 (15) | 23 (19) | 4 (16) | 0.640 |
| Dexamethasone | 330 (95) | 195 (96.5) | 111 (91) | 24 (96) | 0.097 |
| Tocilizumabᵇ | 32 (9.2) | 14 (7) | 18 (15) | 0 | 0.016 |
| Baricitinibᵇ | 83 (24) | 62 (31) | 17 (14) | 4 (16) | 0.002 |

ª Brand name REGEN-COV. Given mainly for moderate or early severe disease or those with immunodeficiency and low antibody levels.

ᵇ Treatments were given for severe patients with rapidly increasing oxygen needs, especially before deteriorating to critical disease.
