# Supplementary material for: Associations of vaccine status with characteristics and outcomes of hospitalized severe COVID-19 patients in the booster era
Source: PLoS One. 2022 May 10;17(5):e0268050. doi: 10.1371/journal.pone.0268050 (PMC9089907; doi:10.1371/journal.pone.0268050)
Supplement: S3 Table — (DOCX) [file pone.0268050.s003.docx]

**Table S3, Maximal values of Selected lab results during hospitalization and correlation to vaccine status.**

|  | Total  n=349  mean ±SD | No vaccine  n=202  mean ±SD | Two Vaccines  n=122  mean ±SD | Booster  n=25  mean ±SD | P |
| --- | --- | --- | --- | --- | --- |
| D-Dimer (mg/L) | 4.86 ± 8.45 | 5.2 ± 8.7 | 4.3 ± 8.2 | 4.3 ± 7.2 | 0.613 |
| Troponin I (ng/L) | 179 ± 1026 | 144 ± 966 | 189 ± 1008 | 404 ± 1485 | 0.489 |
| CPK (U/L) | 454 ± 1676 | 562 ± 2118 | 326 ± 717 | 188 ± 239 | 0.349 |
| LDH (U/L) | 870 ± 965 | 1012 ± 1211 | 687 ± 313 | 562 ± 281 | 0.005 |
| CRP (mg/L) | 146 ±90 | 147 ± 90 | 143 ± 91 | 146 ± 91 | 0.930 |

Abbreviations: CPK, creatine phosphor kinase; LDH, lactate dehydrogenase; CRP, C-reactive protein.
