# Supplementary material for: Associations of vaccine status with characteristics and outcomes of hospitalized severe COVID-19 patients in the booster era
Source: PLoS One. 2022 May 10;17(5):e0268050. doi: 10.1371/journal.pone.0268050 (PMC9089907; doi:10.1371/journal.pone.0268050)
Supplement: S4 Table — (DOCX) [file pone.0268050.s004.docx]

**Table S4, Characteristics of patients by the combined outcome of ICU admission or death.**

|  | Discharged alive and no ICU (n=262)  No. (%) / mean ±SD | ICU admission or deathª (n=87)  No. (%) / mean ±SD | P |
| --- | --- | --- | --- |
| Age>50 years | 212 (81) | 73 (84) | 0.532 |
| Male gender | 154 (59) | 46 (53) | 0.335 |
| HTN | 130 (50) | 56 (64) | 0.017 |
| Hyperlipidemia | 102 (39) | 43 (49) | 0.085 |
| DM | 77 (29) | 35 (40) | 0.061 |
| Obesity (BMI≥30) | 71 (27) | 35 (40) | 0.021 |
| COPD | 33 (13) | 18 (21) | 0.064 |
| IHD | 47 (18) | 21 (24) | 0.206 |
| HF | 36 (14) | 21 (24) | 0.023 |
| AF | 31 (12) | 16 (18) | 0.120 |
| Immuno-deficiency ᵇ | 30 (11.5) | 10 (11.5) | 0.991 |
| CKD | 30 (11.5) | 14 (16) | 0.258 |
| Cognitive decline | 47 (18) | 23 (26) | 0.086 |
| Number of total comorbidities | 2.3 ± 1.9 | 3.1 ± 2.3 | <0.001 |
| Critical disease | 30 (11.5) | 76 (87.4) | <0.001 |
| Hospital duration, days | 5.8 ± 4.9 | 14.9± 12.3 | <0.001 |
| Max D-Dimer (mg/L) | 3.9 ± 7.3 | 8.0 ± 11 | 0.003 |
| Max troponin I (ng/L) | 133 ± 953 | 323 ± 1219 | 0.145 |
| Max CPK (U/L) | 251 ± 401 | 1055 ± 3209 | 0.024 |
| Max LDH (U/L) | 666 ± 259 | 1505 ± 1762 | 0.024 |
| Max CRP (mg/L) | 126 ± 80 | 205 ± 94 | <0.001 |
| Extra–pulmonaryᶜ | 25 (9.5) | 12 (14) | 0.264 |
| AKIᵈ | 41 (16) | 47 (54) | <0.001 |
| Other proven infectionᵉ | 20 (8) | 34 (39) | <0.001 |

Abbreviations: ICU, intensive care unit; HTN, hypertension; DM, diabetes mellitus; BMI, body mass index; COPD, Chronic Obstructive Pulmonary Disease; IHD, ischemic heart disease; HF, heart failure; AF, atrial fibrillation; CKD, chronic kidney disease; CPK, creatine phosphor kinase; LDH, lactate dehydrogenase; CRP, C-reactive protein; AKI, acute kidney injury.

ª Death during hospital stay, ICU admission from the COVID-19 department or directly from the emergency department.

ᵇ Patients included are those with Immunodeficiency secondary to immuno-suppressive therapy or lymphoproliferative malignancy.

ᶜ Cardiovascular, neurological and hematological complications attributed to COVID-19 effect.

ᵈ Acute kidney injury diagnosed as an increase in serum creatinine by ≥0.3 milligrams per deciliter (mg/dl) within 48 hours or an increase in serum creatinine to ≥1.5 times baseline.

ᵉ Non COVID-19 infection is defined by symptoms not attributed to covid-19 with a relevant positive culture.
