# Supplementary material for: Associations of vaccine status with characteristics and outcomes of hospitalized severe COVID-19 patients in the booster era
Source: PLoS One. 2022 May 10;17(5):e0268050. doi: 10.1371/journal.pone.0268050 (PMC9089907; doi:10.1371/journal.pone.0268050)
Supplement: S5 Table — (DOCX) [file pone.0268050.s005.docx]

**Table S5, multivariate analysis of significant risk factors for death or ICU admission in univariant analysis**

| Risk factor | Odds ratio (95% confidence interval) | p Value |
| --- | --- | --- |
| Age under 50 | 0.98 (0.47-2.06) | 0.96 |
| Male gender | 1.15 (0.68-1.97) | 0.6 |
| HTN | 1.36 (0.73-2.52) | 0.33 |
| Hyperlipidemia | 1.07 (0.58-1.94) | 0.84 |
| DM | 1.41 (0.79-2.52) | 0.25 |
| Obesity (BMI > 30) | 1.72 (1.05-2.95) | 0.048 |
| IHD | 1.34 (0.68-2.66) | 0.4 |
| Cognitive decline | 1.61 (0.83-3.11) | 0.16 |
| From symptoms to admission, days | 0.98 (0.92-1.043) | 0.53 |
| Vaccinated with first two doses | 0.64 (0.36-1.14) | 0.13 |
| Vaccinated with booster dose | 0.36 (0.11-1.17) | 0.09 |

Abbreviations: ICU, intensive care unit; HTN, hypertension; DM, diabetes mellitus; BMI, body mass index; IHD, ischemic heart disease;
