# Supplementary material for: Associations of vaccine status with characteristics and outcomes of hospitalized severe COVID-19 patients in the booster era
Source: PLoS One. 2022 May 10;17(5):e0268050. doi: 10.1371/journal.pone.0268050 (PMC9089907; doi:10.1371/journal.pone.0268050)
Supplement: S6 Table — (DOCX) [file pone.0268050.s006.docx]

Table S6, Baseline characteristics and outcomes by SARS-CoV-2 IgG antibody levels among the antibody cohort.

|  | Total n=71 ª | Low sCOVG  (Index level <10)  n=31 | High sCOVG  (Index level >10)  n=40 | P |
| --- | --- | --- | --- | --- |
| Age, mean ± SD, y | 69 ±17 | 74± 14 | 66± 18 | 0.034 |
| Male gender | 46 (65) | 19 (61) | 27 (68) | 0.587 |
| DM | 24 (34) | 9 (29) | 15 (38) | 0.454 |
| Obesity (BMI>30) | 29 (41) | 11 (36) | 18 (45) | 0.418 |
| Immuno-deficiencyᵇ | 16 (22.5) | 10 (32) | 6 (15) | 0.084 |
| Lymphoproliferative disease | 10 (14.1) | 7 (23) | 3 (8) | 0.070 |
| Organ transplantᶜ | 2 (3) | 2 (7) | 0 | 0.103 |
| Immunosuppressive drug | 11 (15.5) | 7 (23) | 4 (10) | 0.146 |
| CKD | 17 (24) | 10 (32) | 7 (18) | 0.148 |
| Number of total comorbidities | 3.24± 2.1 | 3.5± 2.2 | 3.0 ±2.0 | 0.392 |
| Received Casirivimab plus imdevimab ᵈ | 16 (22.5) | 13 (42) | 3 (8) | <0.001 |
| Extra–pulmonary ᵉ | 10 (14) | 8 (26) | 2 (5) | 0.012 |
| Other proven infectionᶠ | 18 (25) | 12 (39) | 6 (15) | 0.023 |

Abbreviations: y, year; DM, diabetes mellitus; BMI, body mass index; CKD, chronic kidney disease.

ª Antibody cohort included only admitted patients after the first two vaccine doses with levels taken earlier than 10 days before first positive PCR swab.

ᵇ Patients included are those with Immunodeficiency secondary to immuno-suppressive therapy or lymphoproliferative malignancy.

ᶜ All patients were kidney transplant recipients.

ᵈ Brand name REGEN-COV. Given mainly for moderate or early severe disease or those with immunodeficiency and low antibody levels.

ᵉ Cardiovascular, neurological, and hematological complications attributed to COVID-19 effect.

ᶠ Non COVID-19 infection is defined by symptoms not attributed to covid-19 with a relevant positive culture.
