# Supplementary material for: Associations of vaccine status with characteristics and outcomes of hospitalized severe COVID-19 patients in the booster era
Source: PLoS One. 2022 May 10;17(5):e0268050. doi: 10.1371/journal.pone.0268050 (PMC9089907; doi:10.1371/journal.pone.0268050)
Supplement: Attachment — Submitted filename: review comments for PONE-D-22-00969.pdf [file pone.0268050.s007.pdf]

This article entitled with “Associations of vaccine status with characteristics and outcomes of hospitalized severe COVID-19 patients in the booster era” compared the characteristics and outcomes in hospitalized severe covid-19 patients among non-vaccine, two vaccines and booster group, which could be an important guidance to better fight COVID-19 together with other clinical studies. As the author mentioned, there were several similar studies published, so this article is a bit lacking in novelty. In addition, I have some minor comments listed below:

1. Only male gender was included in the study, then sexual difference should be discussed.
2. What does the P value stand for in Table 1, 2, S1 and S2, since there were three different groups divided by no vaccine, two vaccines and booster?
