# Supplementary material for: Associations of vaccine status with characteristics and outcomes of hospitalized severe COVID-19 patients in the booster era
Source: PLoS One. 2022 May 10;17(5):e0268050. doi: 10.1371/journal.pone.0268050 (PMC9089907; doi:10.1371/journal.pone.0268050)
Supplement: Attachment — Submitted filename: Response to Reviewers.docx [file pone.0268050.s008.docx]

Linglin Xie,

Academic Editor,
PLOS ONE

3 March 2022

Re: Rebuttal letter for resubmission of manuscript No. PONE-D-22-00969

Dear editor,

We wish to thank you for the opportunity to revise our manuscript, Associations of vaccine status with characteristics and outcomes of hospitalized severe COVID-19 patients in the booster era. We appreciate the in-depth review and constructive suggestions. It is our belief that the manuscript has improved significantly following the changes made.

Revisions in the manuscript are shown using Track Changes. Following this letter are the academic editor and reviewer comments with our responses in italics and reference to the text in red.

In accordance with the reviewers’ suggestions, we have added additional data regarding subgroups, such as patients with previous COVID-19 disease or those received only one dose of the first vaccine. The results section was substantially revised, with further description of our study population, rearrangement of all tables and appropriate citing. We also addressed any grammatical or formatting issues.

We hope that the revisions in the manuscripts and our accompanying responses will be sufficient to make our manuscript suitable for publication in the PLOS ONE journal.

Sincerely,

Ophir Freund, MD

**Academic editor:**

**1. Please ensure that your manuscript meets PLOS ONE's style requirements, including those for file naming.**

*Thank you for all your time and effort. We revised the manuscript to comply with PLOS ONE’s style requirement.*

2. **Please include your full ethics statement in the ‘Methods’ section of your manuscript file. In your statement, please include the full name of the IRB or ethics committee who approved or waived your study, as well as whether or not you obtained informed written or verbal consent. If consent was waived for your study, please include this information in your statement as well.**

*Thank you for this important observation. We included the full name of the IRB in our facility and mentioned the informed consent was waved (2.1 Study design and participants, line 7).*

**3. In your Data Availability statement, you have not specified where the minimal data set underlying the results described in your manuscript can be found. PLOS defines a study's minimal data set as the underlying data used to reach the conclusions drawn in the manuscript and any additional data required to replicate the reported study findings in their entirety.**

**We will update your Data Availability statement to reflect the information you provide in your cover letter.**

*After many deliberations with our institutional review board, the Data Availability statement should be as follows:*

*Due to ethical and privacy concerns the primary dataset cannot be made openly available. The study was done retrospectively and according to the regulations of our institution review board such data could be openly shared. Request for the dataset supporting our results can be made via* [*helsinki@tlvmc.gov.il*](mailto:helsinki@tlvmc.gov.il) *and will be given by the first author after approval.*

4**. Please amend your list of authors on the manuscript to ensure that each author is linked to an affiliation. Authors’ affiliations should reflect the institution where the work was done (if authors moved subsequently, you can also list the new affiliation stating “current affiliation:….” as necessary).**

*We apologize for missing an affiliation and accordingly all authors have a valid affiliation.*

**5. Please include captions for your Supporting Information files at the end of your manuscript, and update any in-text citations to match accordingly. Please see our Supporting Information guidelines for more information:** [**http://journals.plos.org/plosone/s/supporting-information**](http://journals.plos.org/plosone/s/supporting-information)**.**

*We included captions for all supporting files including supplemental tables and raw data.*

**Reviewer comments:**

**Reviewer #1: This article entitled with “Associations of vaccine status with characteristics and outcomes of hospitalized severe COVID-19 patients in the booster era” compared the characteristics and outcomes in hospitalized severe covid-19 patients among non-vaccine, two vaccines and booster group, which could be an important guidance to better fight COVID-19 together with other clinical studies. As the author mentioned, there were several similar studies published, so this article is a bit lacking in novelty.**

*Thank you for your important comment. In our study, we intended to add another proof for the effectiveness of COVID-19 vaccines, which are crucial for ending the current pandemic.*

**In addition, I have some minor comments listed below:**

**1. Only male gender was included in the study, then sexual difference should be discussed.**

*We are sorry for the incoherence of this section in the results. Our study included 200 males which counted for 57% of the study population (see table 1 and first paragraph under Results). We included only patients with severe disease which it might explain the male predominance, as it is a risk factor for severe COVID-19^1,2^. We also changed our description to include the percentage of females in our cohort (line 3 in General characteristics and outcomes section).*

2**. What does the P value stand for in Table 1, 2, S1 and S2, since there were three different groups divided by no vaccine, two vaccines and booster?**

*Thank you for this important inquiry. Our statistical team performed analysis for the three groups using Chi-Square tests and ANOVA tests. The P value between the three groups represent a significant difference in the results between the groups. The P value does not indicate which group had a higher or lower result and is not related to any specific group. In the article, we only state for each variable if it was significantly different between the groups (based on the p value), while any trend (such as lower occurrence of ICU admission in vaccinated patient) is described based on its frequency in each group.*

**Reviewer #2: In this manuscript, Freund et al. identified and summarized characteristics of outcomes of COVID-19 patients according to their vaccine status (grouped into three) during the fourth wave in Israel. The association of vaccine status with outcomes of hospitalized patients is well put and studied. However, I have some concern about the design of study, especially the categorization of patient groups, which also leads to the obvious limitation of the study due to relatively low number of patient samples from the booster group, although the statistical methods are all properly implemented.
In addition, there are some big grammatical and formatting issues throughout the manuscript. Please make significant efforts to correct and proofread it before re-submission. Comments are listed below.

Major**
**--What biases/differences would it present to the study if the authors single out patients that were vaccinated only once? (i.e., first dose only, without the second dose). This would lead to four groups: Nonvaccinated (zero dose) vs. one dose vs. two doses (fully vaccinated) vs. booster dose. I believe it would strengthen the design of this study.**

*We wish to thank you for the time and effort reviewing our manuscript. This is an important issue that was not addressed by us in the article. As you mentioned and was also stated in our methods section, patients who were vaccinated only once were included in the non-vaccinated group. In Israel, vaccinations were under the responsibility of the health maintenance organizations (HMOs) which resulted in a remarkably high compliance of the public. In our study, only two patients received the first vaccine dose without the second. Taking that into consideration, separating this group from others was not possible. Both patients had the first vaccine dose more than 6 months before their admission, a period which was previously shown to have reduced immunity effect by the vaccines^3,4^, leading us to include them in the non-vaccinated group. We included this explanation in the methods (lines 13-15 under the Definitions section).*

--“**Tocilizumab and baricitinib were given mostly for critical COVID-19 disease and were used in 9.2% and 24% of the patients, respectively”. This is helpful, but what is the exact ratio/percentage of the usage of these two in the three different groups, respectively?**

*The number of patients received each of these treatments appear in table S2 in the appendix, including percentages. Following your comment, we included a more detailed description of this issue in the text (lines 14-20 under General characteristics and outcomes).*

**--There is no clear explanation or descriptive summary based on the results of Table 1**.

*Thank you for this observation. Descriptive summary based on the results of Table 1 appears under General characteristics and outcomes (section 3.2, a page before the Table in the manuscript submitted). Accordingly, we changed the description and added additional major results from Table 1.*

-- **Higher level of oxygen support was inversely proportional to the number of vaccines given (p = 0.004). Is this referring to Table 2? Please cite properly. Also, this s entence is a bit confusing. When you state two variables to be inversely proportional, avoid using comparative forms.**

*We agree with your remarks and regret not citing appropriately. Accordingly, we rephrased the sentence and change its location to appear as part of the results of Table 2 under the section “Disease outcomes and comparison by vaccine group” (line 3 section 3.3). Following your valuable remarks, we reorganized the entire results section as explained under the next comments.*

**--Please cite the tables in the correct order as in which they appear in the manuscript. Citations in the results sections seem very disorganized. It seems messed up in the results section. Also, where would you cite Table S2? In addition, Table S2 row 3 is poorly formatted.**

*Thank you for your influential remarks regarding this topic. We rearranged the entire results section. Currently, each table is cited only in one separate section. As a result, all tables are cited according to the order of their appearance. Table S2 is now S3 (based on its appearance) and described under “Disease outcomes and comparison by vaccine group” (section 3.3 under Results, lines 9-12). We changed Table S2 in the supplement to the appropriate format.*

--**Given the limitation of the study, especially the small number of patients after the booster shoot, I do not find strong implications for this design. The ratio between the two groups (no vaccine vs two vaccines) is acceptable, and it could even be improved by taking the suggestion from my first comment. However, such a low patient number for booster group seems like a concern to me.**

*We understand your concern regarding the small sample of patients after the booster Vaccine. We believe that the strength of our study as well as its uniqueness is by including only severe COVID-19 patients that were hospitalized because of their COVID-19 disease. Fortunately, the booster vaccine is highly effective in preventing severe disease and hospitalizations. In two main studies, the booster vaccine lowered the rate of severe disease by a factor of 19.5 and demonstrated a 94% effectiveness to prevent COVID-19 associated emergency department hospitalizations^5,6^. In a multicenter study, describing severe COVID-19 patients, only 4% of the admitted patients had the booster vaccine^7^. The above results are in accordance with the smaller sample of patients after the booster vaccine, compared with the other two groups in our study.*

**--In addition to my last comment, does any of the group include patients who got reinfection?**

*Only one patient had a prior COVID-19 disease. For this reason, we decided not to address it separately and he was included in the non-vaccinated group based on his vaccine status. Following your important remark, we added these details to the text (Methods, under “Definitions” lines 14-15).*

Minor
**Grammatical: “clinical information and laboratory results were obtained by reviewing each patient medical electronic records”. Please rephrase correctly.
Grammatical: “Patients were defined as critically ill if met any of the following criteria:” Please rephrase.
Formatting: “Patients presented with deteriorating respiratory distress e.g., rapidly increasing oxygen demands”, format the use of e.g., properly.
Grammatical: “During the study period 436 patients were admitted to our center with active COVID-19, of them 349 met the definition of severe or critical disease and were included in the study.” Please rephrase.**

*We agree with all your above comments and rephrased or changed the sentences accordingly.*
**Please include table caption above the table instead of placing it underneath**.

*We placed all captions above the tables.*

1.	Lewnard JA, Liu VX, Jackson ML, et al. Incidence, clinical outcomes, and transmission dynamics of severe coronavirus disease 2019 in California and Washington: Prospective cohort study. *BMJ*. 2020;369(9). doi:10.1136/bmj.m1923

2.	Cummings MJ, Baldwin MR, Abrams D, et al. Epidemiology, clinical course, and outcomes of critically ill adults with COVID-19 in New York City: a prospective cohort study. *Lancet*. 2020;395(10239):1763-1770. doi:10.1016/S0140-6736(20)31189-2

3.	Thomas SJ, Moreira ED, Kitchin N, et al. Safety and Efficacy of the BNT162b2 mRNA Covid-19 Vaccine through 6 Months. *N Engl J Med*. 2021;385(19):1761-1773. doi:10.1056/nejmoa2110345

4.	Goldberg Y, Mandel M, Bar-On YM, et al. Waning Immunity after the BNT162b2 Vaccine in Israel. *N Engl J Med*. Published online 2021:1-10. doi:10.1056/nejmoa2114228

5.	Thompson MG, Natarajan K, Irving SA, et al. Effectiveness of a Third Dose of mRNA Vaccines Against COVID-19-Associated Emergency Department and Urgent Care Encounters and Hospitalizations Among Adults During Periods of Delta and Omicron Variant Predominance - VISION Network, 10 States, August 2021-January 2022. *MMWR Morb Mortal Wkly Rep*. 2022;71(4):139-145. doi:10.15585/mmwr.mm7104e3

6.	Bar-On YM, Goldberg Y, Mandel M, et al. Protection of BNT162b2 Vaccine Booster against Covid-19 in Israel. *N Engl J Med*. 2021;385(15):1393-1400. doi:10.1056/nejmoa2114255

7.	Uzun O, Akpolat T, Varol A, et al. COVID-19: vaccination vs. hospitalization. *Infection*. Published online January 2022:1-6. doi:10.1007/s15010-021-01751-1
